# Characterization of *Globodera ellingtonae* Populations from Chile Utilizing Whole Genome Sequencing

**DOI:** 10.21307/jofnem-2021-088

**Published:** 2021-11-03

**Authors:** C.N. Hesse, I. Moreno, O. Acevedo Pardo, H. Pacheco Fuentes, E. Grenier, L. M. Dandurand, I. A. Zasada

**Affiliations:** 1 USDA ARS Horticultural Crops Research Unit, Corvallis, OR 97330; 2 Servicio Agrícola y Ganadero (SAG). División de Protección Agrícola y Forestal y Red SAG de Laboratorios, Santiago, Chile; 3 IGEPP, INRAE, Agrocampus Ouest, Université Rennes 1, 35650, Le Rheu, France; 4 875 Perimeter Drive MS 2329, University of Idaho, Moscow, ID 83844-2329

**Keywords:** Genome, *Globodera ellingtonae*, Population genetics, Potato cyst nematode

## Abstract

*Globodera ellingtonae* was originally described from populations collected in the United States. In the original description, ribosomal DNA loci from *Globodera* sp. collected in Chile and Argentina were similar to *G. ellingtonae*, suggesting this nematode originated in this region of South America. In an effort to find additional populations of *G. elllingtonae,* collection trips were conducted in 2017 and 2020 in the Antofagasta and Arica y Parinacota Regions in Northern Chile, respectively. *Globodera* sp. were more prevalent in Antofagasta (17 samples collected, 53% positive for *Globodera* sp.) than in Arica y Parincota (16 samples collected, 13% positive for *Globodera* sp.). The genomes of single cysts (N ≥ 3) from four fields were sequenced. Additionally, the genomes of the *G. ellingtonae* population from Oregon and a *Globodera* sp. population originally collected in Antofagasta Region but maintained in culture in France were also sequenced. Based upon a HSP90 sequenced data mined from WSG data, all of the populations from the Antofagasta Region were *G. ellingtonae* and grouped in a monophyletic clade. A population collected from the Arica y Parincota Region was identified as *G. rostochiensis* based upon HSP90 data. Genome-wide SNP patterns of the *G. ellingtonae* populations showed strong clustering based on geographic location indicating that *G. ellingtonae* has high genetic diversity within Chile. A phylogenetic tree derived from 168,354 binary SNPs in the nuclear genome showed separate but distinct clustering of the Oregon population and the population from Antofagasta maintained in France. The Oregon *G. ellingtonae* population subtended the Chilean clades and placed on a long branch representing approximately twice the genetic variation observed among all Chilean *G. ellingtonae* populations. The possibility remains that *G. ellingtonae* from Chile may be sufficiently diverged to constitute a new species from *G. ellingtonae* originally described from a population collected in Oregon.

*Globodera ellingtonae* (Handoo et al., 2012) was first detected, and subsequently described from populations collected in Oregon and Idaho (Handoo et al., 2012). The ability of *G. ellingtonae* to reproduce on potato and tomato has been demonstrated (Lax et al., 2014; Zasada et al., 2013). The biology of this nematode has been characterized and many of the biological characteristics indicate that it behaves more similarly to *G. rostochiensis* than to *G. pallida*. *Globodera ellingtonae* appears to have similar temperature requirements for hatch and development as *G. rostochiensis* (Phillips et al., 2015). Potatoes that carry the H1 gene which confers resistance to *G. rostochiensis* are also resistant to *G. ellingtonae* (Zasada et al., 2013). However, unlike the potato cyst nematodes *G. rostochiensis* and *G. pallida*, *G. ellingtonae* has not been shown to be a strong pathogen to potato in experiments conducted in Oregon (Zasada et al., 2019).

The initial molecular analysis of *G. ellingtonae* from Oregon showed that the population was distinct from *G. pallida*, *G. rostochiensis*, and *G. tabacum* (Skantar et al., 2011). In this same study, a phylogenetic analysis based on the ITS1 and -2 rDNA indicated that the *G. ellingtonae* population from Oregon was most similar to *Globodera* sp. populations collected in Argentina and Chile. A subsequent study on a *G. ellingtonae* population from Argentina obtained similar results based on HSP90 and ITS regions (Lax et al., 2014). Additionally, in these analyses *G. ellingtonae* was more closely related to *G. rostochiensis* and the *G. tabacum* complex than to *G. pallida*. A multigene phylogeny based upon 6,933 bp encompassing 11 genes, further confirmed the closer relationship of *G. ellingtonae* to *G. rostochiensis* and the *G. tabacum* complex than to *G. pallida* (Zasada et al., 2015).

To date, there are sequences for four populations of *G. ellingtonae* available in Genbank from the United States, Chile, and Argentina (Handoo et al., 2012; Lax et al., 2014; Skantar et al., 2011). This is just a handful of data compared to what is available for *G. rostochiensis* and *G. pallida* (Subbotin et al., 2020). To expand our knowledge of the biodiversity of *G. ellingtonae* and facilitate further work on *Globodera* biogeography additional molecular data is required. In this study, we performed whole genome shotgun (WGS) sequencing on *G. ellingtonae* populations collected from Chile. Whole genome sequence data enables genome-wide comparisons of closely related populations and provides an increased ability to discern population structure beyond what is possible using marker loci (i.e. ITS and HSP90). Utilizing WGS data, we then explored population genetics among the *G. ellingtonae* populations and compared informative gene regions of these populations to available data.

## Materials and methods

### Collection of *Globodera* populations

In April 2017, a collection trip was undertaken in the Antofagasta Region of Chile. Agricultural fields in Socaire, Camar, Talabre, Caspana, and Toconce were sampled. Another collection trip was conducted in March 2020 in the Arica y Parinacota Region. Agricultural fields in Azapa, Chapiquiña, Pachama, Belén, Putre, Visvíri, and Socoroma were sampled. The fields were either fallow or under production with a diversity of crops including potato, tomato, maize, chard, and quinoa. Seventeen soil samples were collected in 2017 and 16 in 2020.

To collect samples, a trowel or probe (2.5 cm diameter) was used and soil was collected from multiple locations within a field to a depth of 10 to 15 cm and combined. The soil was placed in a paper bag. At the Servicio Agrícola y Ganadero Nematology Laboratory (Santiago, Chile) soil samples were placed on trays and allowed to dry for at least a week. Cysts were then extracted from 250 g of soil using a modified Fenwick can (Fenwick, 1940). Cysts were picked from samples and placed in DESS (Yoder et al., 2006). Samples were shipped to USDA-ARS Horticultural Crops Research Unit in Corvallis, Oregon for sequencing.

### Sequencing and analysis of *Globodera* populations

Four of the Chilean *Globodera* sp. populations were selected for sequencing. These were chosen because enough cysts were available, and cysts contained abundant eggs. These populations were from Putre, Talabre, and Socaire. The two populations from Socaire were collected from the same field but on different dates (B was collected on 23 October 2017 and H was collected on 25 April 2017). Cysts for sequencing were also obtained from a culture of *Globodera* sp. originally collected Calama, Chile in the Antofagasta Regoin and maintained on potato at Institut National de la Recherche Agronomique (INRAE; Rennes, France). The *G. ellingtonae* population from Powell Butte, Oregon, which was used for the original description of the species (Handoo et al., 2012), was also sequenced. From each population at least three individual cysts were selected for sequencing. In total, sequences from 23 individual cysts representing five distinct geographic populations were used in the final analysis.

Genomic DNA was obtained from individual cysts and eggs contained within using the QIAamp DNA Micro kit (Qiagen, Germantown, MD) following the manufacturer instructions for isolation from tissues. Additional cell wall disruption was done during the second step of the QIAamp protocol wherein sterile plastic pestles were used to crush cyst tissue and mechanically lyse eggs. Extracted DNA was sent to the Core Services Lab at the Center for Genome Research and Bioinformatics (CGRB) at Oregon State University (Corvallis, OR) for Illumina library generation using the Nextera XT preparation kit (Illumina, San Diego, CA). Each sample was uniquely barcoded and multiplexed in equimolar concentrations prior to sequencing on the Illumina HiSeq 3000 platform. Paired-end 150 bp reads were obtained from the CGRB along with associated sequencing quality information. Raw sequence data is available for download from the Sequence Read Archive (SRA) under BioProject PRJNA748409.

Visual inspection of sequencing quality reports resulted in the removal of one sample (Socaire B1) from additional analyses based on the overall poor sequencing quality metrics. The remaining samples were trimmed to remove bases with Phred quality scores < 20 and remove adapter sequences using bbduk (https://sourceforge.net/projects/bbmap/).

A single-gene phylogeny was constructed for Heat Shock Protein 90 (HSP90) to facilitate comparison to other *Globodera* sequences available in Genbank. A single 1402 bp HSP90 sequence from *G. ellingtonae* (MK105568.1) was used as a reference to anchor the assembly of the locus from each sample using the software package SAUTE (Souvorov and Agarwala, 2021). Sequences from identified *Globodera* and *Cactodera* (outgroup) from PopSet 1780190675 (Skantar et al., 2021) were used for comparison, as were the partial HSP90 sequences obtained by Lax et al. (2014) from a *G. ellingtonae* population collected in Argentina. All sequences were aligned using Muscle v3.8.31 (Edgar, 2004), trimmed to the shortest sequence length (final alignment of 437 bp) and a maximum likelihood phylogenetic tree was constructed using FastTree v2.1.11 (Price et al., 2010). The HSP90 locus could not be reliably reconstructed for three samples: Talabre D7, Socaire B7, and Socaire H6, therefore, these samples are not included in the HSP90 phylogenetic tree.

The publicly available *G. ellingtonae* genome assembly, GCA_001723225.1 (Phillips et al., 2017), was used as the reference for nuclear genome variant determination. Mitochondrial variants were determined using the reference assembly KU726971 and KU726972, corresponding to the large- and small-circle mtDNA genome (Phillips et al., 2016). Quality filtered reads were mapped to the reference genome using BWA (Li and Durbin, 2009) prior to variant calling in Freebayes (Garrison and Marth, 2012). The resulting variant call file was filtered using VCFtools (Danecek et al., 2011) and BCFtools (Li et al., 2009) to remove indels and retain only single and multiple nucleotide polymorphisms with minimum quality > 30 and per-sample read depth between 3 and 100. Variant calls were imported into the statistical software R (R Core Team, 2021) using the “vcfR” (Knaus and Grünwald, 2017), “poppr” (Kamvar et al., 2014), and “adegenet” (Jombart, 2008) packages for further analysis. Unweighted Pair Group Method with Arithmetic Mean (UPGMA) trees for both nuclear and mitochondrial genomes were generated from 168,354 and 59 binary SNPs, respectively. Due to the nature of the SNP calling method based of a reference genome, the inclusion of an outgroup from another *Globodera* species was not possible. As such, the phylogenetic trees were rooted at the mid-point for easy visualization

### Morphometrics of *Globodera* populations

Measurements for the *Globodera* sp. population from the Antofagasta Region in Chile maintained by INRAE were made by Dr. D. Mugniery in 2007-2008. Second-stage juvenile (J2) measurements included (*n* = 32): body length, stylet length, tail length, and hyaline length all in µm. Cyst measurements included (*n* = 29): fenestra length (µm), distance from anus to nearest edge of fenestra (µm), number of cuticular ridges between vulva and anus, and Granek’s ratio (Hesling, 1973). Additionally, individual *G. ellingtonae* J2 (*n* = 22) and cyst (*n* = 4) morphometric data collected in 2008 was obtained from Dr. Z. Handoo (USDA-ARS Beltsville, MD). Mean (minimum and maximum) *G. ellingtonae* morphological data from Argentina was also considered (Lax et al., 2014). A character phylogeny was constructed in Mesquite (Maddison and Maddison, 2019) from the morphometric attributes for which complete data existed in all populations. When only a range of measurements was available for a given population, synthetic individuals were generated by randomly sampling characters within the measurement range.

## Results

### Occurrence and population densities of *Globodera* sp. in Northern Chile

**Figure 1:**
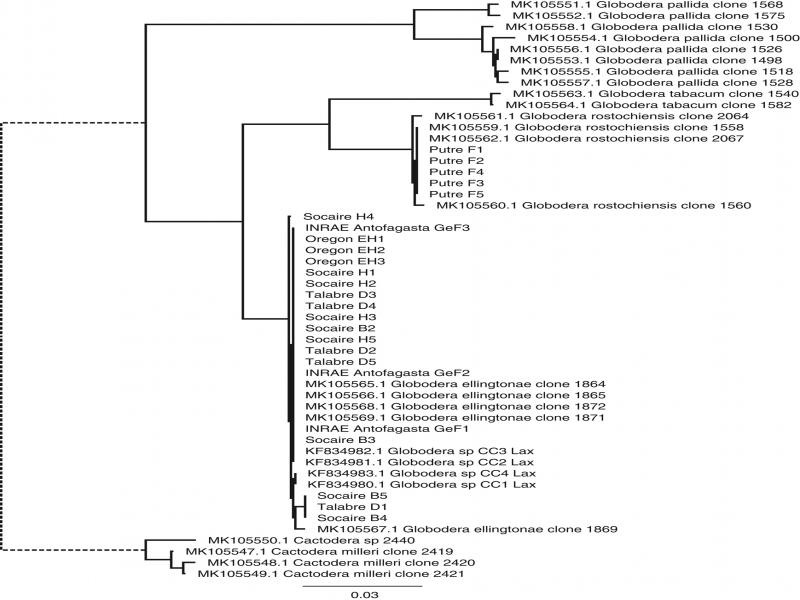
Single gene phylogeny of Heat Shock Protein 90 (HSP90) constructed form a 437 nt alignment of morphologically identified *Globodera* spp. from Skantar et al. (2021), *G. ellingtonae* from Argentina (Lax et al., 2014), and populations from this study. The maximum likelihood tree was rooted to the outgroup *Cactodera* and the branch of the outgroup was artificially truncated for clarity (dashed lines).

*Globodera* sp. cysts were recovered from 9 out of 17 of the samples collected in 2017 from the Antofagasta Region. Cyst densities ranged from 2 to 50 cysts/250 g soil. These populations were collected from Socaire, Talabre, and Toconce. In 2020, only 2 samples out of 16 (13%) had cysts in the Arica y Parinacota Region. Population densities of these samples were 10 and 60 cysts/250  g soil; both samples were collected from Putre. The populations collected from the Arica y Parinacota Region were morphologically identified as *G. rostochiensis* (data not shown). This identification was further confirmed in the HSP90 phylogeny which showed distinct grouping of the Putre population with identified *G. rostochiensis* populations (Fig. 1).

### Single Locus HSP90 phylogeny

The nuclear HSP90 locus was used to facilitate comparison of *G. ellingtonae* sequences from this study to those of Lax et al. (2014). The inclusion of the Argentinian *G. ellingtonae* population allowed for direct comparison with the *G. ellingtonae* populations from Chile. The partial HSP90 amplicons available for the Argentinian *G. ellingtonae* population restricted the analysis to a 437 nucleotide alignment with limited sequence variation. As a result, the HSP90 phylogeny did not differentiate *G. ellingtonae* populations with only three nucleotide polymorphisms separating all populations within the clade (Fig. 1).

### Genomic characterization

**Figure 2:**
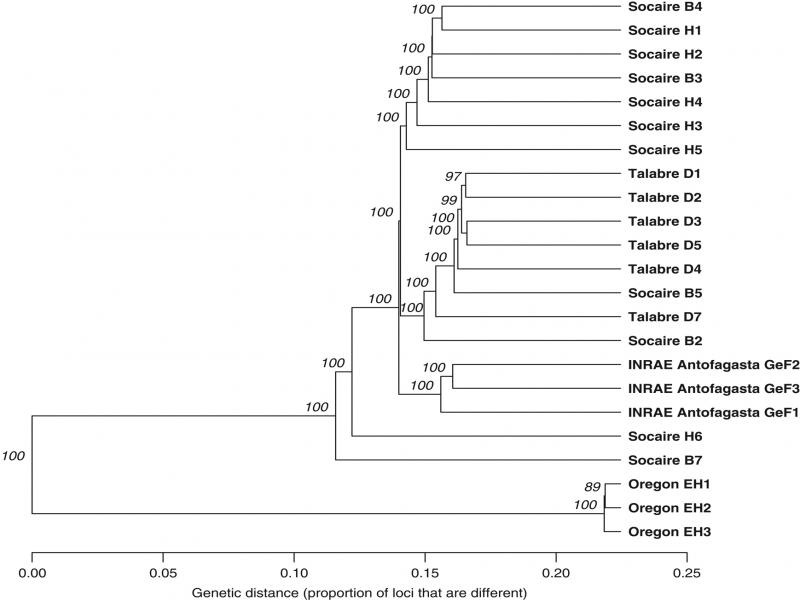
Nuclear single nucleotide polymorphism (SNP) phylogenetic reconstruction of 23 *Globodera ellingtonae* cysts from Chile, United States, and a cultured population originally collected in Chile and maintained at Institut National de la Recherche Agronomique (INRAE; Rennes, France). Phylogenetic relationships were inferred using Unweighted Pair Group Method with Arithmetic Mean (UPGMA) clustering from a bitwise distance matrix derived from 168,354 binary SNPs across the nuclear genome.

As stated above, the population from Putre was identified as *G. rostochiensis*, therefore it was not included in further genomic characterization. A total of five *G. ellingtonae* populations were included in the analyses. Whole-genome shotgun sequencing followed by genetic variant calling of individual cysts revealed a population structure broadly correlated with geographic region. A phylogenetic tree derived from 168,354 binary SNPs in the nuclear genome showed separate but distinct clustering of the INRAE Antofagasta and Oregon *G. ellingtonae* populations (Fig. 2). Seven cysts from Socaire formed a well-supported clade, as did cysts from Talabre which formed a supported clade along with two SocaireB cysts. The relationship among the primary *G. ellingtonae* Talabre, Socaire, and INRAE Antofagasta clades remained unresolved despite the large number of loci used in the phylogeny. Cysts from the *G. ellingtonae* Oregon population subtended the Chilean clades and placed on a long branch representing approximately twice the genetic variation observed among all Chilean populations.

Chilean *G. ellingtonae* populations, along with the INRAE Antofagasta population, exhibited long terminal branch lengths suggesting relatively high genetic differentiation among cysts from the same field. In contrast, cysts from the Oregon population of *G. ellingtoane* were very similar to one another with comparatively short terminal branch lengths. The placement of the INRAE Antofagasta *G. ellingtonae* population was interleaved with Chilean populations, reflecting the original provenance of the cultured population within the same sampling region as the *G. ellingtonae* populations collected in 2017.

**Figure 3:**
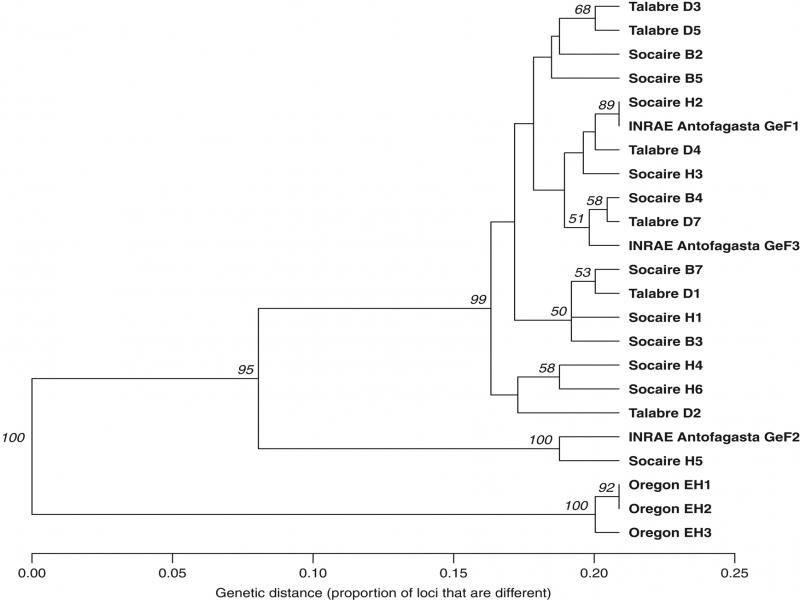
Mitochondrial single nucleotide polymorphism (SNP) phylogenetic reconstruction of 23 *Globodera ellingtonae* cysts from Chile, United States, and a cultured population originally collected in Chile and maintained Institut National de la Recherche Agronomique (INRAE; Rennes, France). Phylogenetic relationships were inferred using Unweighted Pair Group Method with Arithmetic Mean (UPGMA) clustering from a bitwise distance matrix derived from 59 binary SNPs across the nuclear genome.

Mitochondrial variants totaled 59 binary SNPs across all *G. ellingtonae* populations and the corresponding phylogeny was largely unresolved (Fig. 3). Distinct clustering of the Oregon *G. ellingtonae* population reflected the same subtending placement to the Chilean and INRAE Antofagasta *G. ellingtonae* populations as the genomic SNP phylogeny (Fig. 2). The relationships among *G. ellingtonae* Chilean and INRAE Antofagasta populations were unresolved as most branching had poor bootstrap support. Like the nuclear SNP phylogeny, the Oregon *G. ellingtonae* population had characteristically short terminal branches while the Chilean and INRAE Antofagasta *G. ellingtonae* populations had comparatively long branches.

### Morphological comparison

The measurements of J2 and cysts of *G. ellingtonae* populations from Chile, United States, and Argentina did not resolve in the character phylogeny (data not shown). Limited morphological data was available for some populations thereby restricting the number characters available to use in a character phylogeny. Of the characters available, all displayed overlapping ranges with no discriminatory power among populations. A summary of morphological measurements of *G. ellingtonae* populations reported in the literature (Handoo et al., 2012; Lax et al., 2014) and the new measurements from the *G. ellingtonae* population maintained at INRAE are in Table 1.

**Table 1.** Morphometric characters of second-stage juveniles and cysts of *Globodera* sp. from Oregon, Argentina, and Antofagasta, Chile. Measurements are in µm and are mean + standard deviation (minimum – maximum).

	Second-stage juveniles				Cysts			
Population	Body length	Stylet length	Tail length	Hyaline length	Diameter of vulval basin	Distance from anus to vulval basin	No. cuticular ridges	Granek’s ratio
*G. ellingtonae* (Oregon)^a^	450 + 28 (365-515)	20.9 + 0.9 (19-22.5)	46.7 + 3.5 (39-55)	24.3 + 2.7 (20-32.5)	27.4 + 5.4 (20-42.5)	64.5 + 10.3 (50-85)	13 + 2.3 (10-18)	2.4 + 0.4 (1.7-3)
*G. ellingtonae* (Argentina)^b^	458 + 23 (418-526)	23 + 0.6 (22-24)	50 + 3 (43-56)	25 + 3 (19-31)	23 + 5 (12-36)	52 + 12 (32-84)	13 + 4 (8-25)	2.6 + 0.9 (0.9-5.9)
*Globodera* sp. (Antofagasta)^c^	490 + 22.5 (447-533)	21.9 + 0.8 (20-23.2)	49.2 + 3.1 (43-54.8)	24 + 2.6 (18.5-29.5)	21 + 2.5 (15.5-25.1)	68.1 + 14.8 (43-115.6)	13.8 + 3.4 (7-20)	3.3 + 0.9 (2-5.8)

Notes: ^a^Measurements based on 93-106 second-stage juveniles and 22-23 cysts (Handoo et al., 2012).

^b^Measurements based on 37-41 second-stage juveniles and 42 cysts (Lax et al., 2014).

^c^Measurements based on 32 second-stage juveniles and 29 cysts. Measurements were made by Dr. D. Mugniery in 2007-2008.

## Discussion

This study expands the documented biodiversity of *Globodera* spp. in their native range, enabling comparative studies within the species and among congeneric species. The use of WGS sequencing data represents a shift from traditional single- or multi-locus comparisons to genome-wide contrasts. While multi-gene comparisons adequately delineate species, they often lack the necessary resolution for within-species or population level comparisons. As such, integration of these WGS data with previous studies of *Globodera* biodiversity is difficult. Traditional phylogenetic marker loci were often selected because they are amenable to reliable Sanger sequencing due in part to their multi-copy nature (rDNA) or presence on the highly abundant mitochondrial genome (COI and cytB). In contrast, these attributes are detrimental to *de novo* assembly as within-genome copy variation often confounds assembly methods (Souvorov and Agarwala, 2021) resulting in fragmented or missing loci. Despite this limitation we were able to construct an HSP90 phylogeny from our sequence data using targeted assembly of the single-copy nuclear gene thereby allowing comparison to previously published work. Based upon the HSP90 phylogeny, all of the populations collected in the Antofagasta Region of Chile would be considered *G. ellingtonae*.

From the WGS data, genome-wide SNP patterns showed strong clustering of *G. ellingtonae* populations based on geographic location indicating the species has high genetic diversity within Chile. Furthermore, the distal placement of the Oregon population suggests the original introduction source to the United States was not closely related to any of the populations collected in Chile nor the cultured INRAE Antofagasta population. This observation would not have been evident if only the single-locus HSP90 gene was considered. Sequence data for *G. ellingtonae* isolated from Argentina is available for a fragment of the HSP90 locus (Lax et al., 2014). Alignment of these Argentinean sequences with HSP90 locus from this study did not provide sufficient sequence variability for comparison. Further sampling within the native range of *G. ellingtonae*, including Argentina and Bolivia, as well as additional locations with Chile, will be necessary to identify the source *G. ellingtonae* population of the U.S. introduction.

Similar to patterns observed by Dr. E. Grenier (personal communication) using microsatellite data, the whole-genome SNP data revealed that the genetic diversity in the *G. ellingtonae* Oregon population was much lower than that observed in *G. ellingtonae* natural populations from Chile and the cultured INRAE Antofagasta population. This finding reflects the presumed genetic bottleneck expected from an accidental introduction, such as the case in Oregon. High genetic diversity within South American *Globodera* populations has been documented previously (Picard et al., 2004; Subbotin et al., 2020; Thevenoux et al., 2020). In fact, the cysts found under a single plant often show the same diversity as the cysts collected in the entire field (Picard et al., 2004), likely due to the high occurrence of multi-paternal cysts (Grenier et al., 2016). The INRAE Antofagasta *G. ellingtonae* population maintained in culture displayed similarly high genetic diversity as the populations collected directly from the field in Chile, presumably due to rearing methods that aimed to maintain genetic diversity throughout time. In contrast, the accidental introduction of *G. ellingtonae* cysts to Oregon likely resulted in few cysts distributed over a large proximity in less-than-ideal conditions resulting in low reproduction and limited outcrossing.

Chronis et al. (2014) developed a taqman qPCR assay to differentiate *G. ellingtonae* from other *Globodera* species utilizing a conserved 10 bp deletion in the chorismate mutase (CM) gene. Initial assessment of the INRAE Antofagasta population by Chronis et al. (2014) with the CM TaqMan assay indicated the population was not *G. ellingtonae*. However, based on our sequencing data, nucleotide alignment of the probe site for the cultured population showed the conserved deletion is present as are the forward and reverse priming sites suggesting the TaqMan assays should classify the population as *G. ellingtonae*; this is further supported by the HSP90 phylogeny. The Oregon population also possess the discriminatory deletion however the sequence similarity with the other populations across the entire 2kb CM alignment was only 98.5%. The possibility remains that the *G. ellingtonae* population from Chile may be sufficiently diverged from *G. ellingtonae* populations from Oregon to constitute a new species. Further sampling of *Globodera* in southern South America is warranted to understand the genetic diversity of *G. ellingtonae* and the relationship to the Oregon population. Additionally, the use of WGS to explore genomic difference among *Globodera* populations will become more valuable as more plant-parasitic nematode genomes become available.
